# Wild-AC: A fast, index-free multi-pattern string matching algorithm with wildcard support for proteomics

**DOI:** 10.1186/s12859-026-06686-8

**Published:** 2026-10-09

**Authors:** Chris Bielow

**Affiliations:** Institut für Mathematik und Informatik, Freie Universität Berlin, Takustr. 9, 14195 Berlin, Germany

**Keywords:** String matching, Multi-pattern, Aho Corasick, Wildcard, FM-index

## Abstract

**Background:**

Efficient multi-pattern string matching with wildcard support is essential in proteomics, where peptides must be mapped to protein databases containing ambiguous amino acids (e.g., *B*, *Z*, *X*). Existing approaches either require preprocessing (e.g., FM-index) or fail to handle wildcards in the text.

**Results:**

We present Wild-AC, a modified Aho-Corasick algorithm that supports wildcards in the text by branching the search into parallel ‘scout’ paths, one per possible wildcard representation, while the unmodified primary search continues unaffected in the common, wildcard-free case. Wild-AC outperforms the FM-index (wildcard case) and matches or exceeds Wu-Manber (exact search) in speed for realistic proteomics workloads, i.e., 1000–500 000 peptide patterns (average length ∼18 amino acids) searched against protein databases (texts) of 3 × 10^6^–2.1 × 10^8^ characters. On databases masked to a wildcard rate of 5%, Wild-AC retains its advantage for large peptide sets, while the FM-index becomes preferable for small ones. It requires no index, scales well with pattern count, and supports multi-threading. The C++ implementation is open source at https://github.com/Wild-AC/Wild-AC.

**Conclusion:**

Wild-AC extends the Aho-Corasick algorithm to support wildcards and outperforms FM-Index and Wu-Manber in the task of peptide-protein mapping while considering ambiguous amino acids.

**Graphical abstract:**

**Figure:**
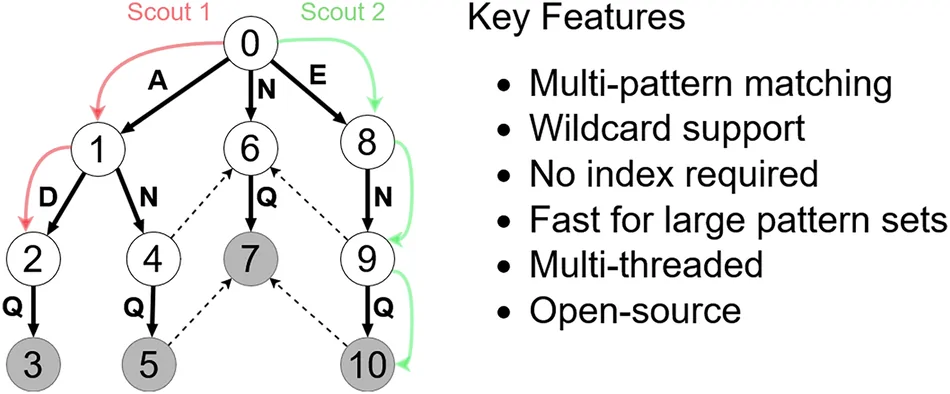

## Background

Algorithms for string matching, in both exact and approximate flavors, are extensively researched areas in computer science and bioinformatics. The literature offers a plethora of solutions to find approximate matches for a given pattern in a database. See Navarro & Raffinot [1] for a broad introduction. In this paper, we introduce our new algorithm, Wild-AC, which is a multi-pattern string matching algorithm with support for wildcards, based on the Aho-Corasick (AC) algorithm [2]. While approximate string matching for a single pattern has been extensively studied, the case of multiple patterns remains less explored. Most existing work on Hamming distance allows wildcards only in patterns, not in the text. In this work, wildcards are allowed in the text (e.g., a protein sequence), not in the patterns (e.g., a peptide sequence). Conceptually, Wild-AC’s core idea is simple: it runs the unmodified AC automaton on the text and, whenever a wildcard character is encountered, bifurcates the search by branching off a parallel ‘scout’ search path for every character the wildcard could represent, while the primary search path continues unaffected; scouts are pruned as soon as they can no longer contribute a hit not already found by the primary or an ancestor scout (see Section 2.2).

We will show that Wild-AC is highly competitive against state-of- the-art algorithms (such as the FM-index [3]), while at the same time not requiring any preprocessing of the text (i.e., no index generation), as well as a relatively simple implementation.

String matching algorithms can be divided into online (no preprocessing) and offline methods, where offline typically involves preprocessing the text to create a database/index of some kind. Algorithms for single patterns which do not preprocess the text (so called ‘online algorithms‘), such as the Bitap algorithm [4](also known as Shift-Or/Shift-And) and its extensions which can cope with approximate matches, are usually not effective for multiple patterns, since the text needs to be scanned many times. Shift-And in particular has a multi-pattern extension, but it is only efficient as long as the summed length of all patterns does not exceed the machine’s register size (e.g., 32, 64 or 128 bits), which is far below the pattern counts typical of proteomics (see below). Fortunately, for most other online algorithms there are dedicated multi-pattern variants which scan the text only once for all patterns simultaneously, such as Aho-Corasick [2] (a multi-pattern generalization of the Knuth- Morris-Pratt automaton), Wu-Manber [5] and Commentz-Walter [6] (generalizing Boyer-Moore), Set-Horspool [1] (generalizing Horspool) and Multi-BNDM [7] (generalizing BNDM). However, most of them shine only when the number of patterns is small (on the order of tens to a few hundred) and quickly deteriorate in performance otherwise. Notable exceptions are Wu-Manber and the Aho-Corasick (AC) algorithms, which handle an increasing number of patterns very favorably. AC is used in network intrusion detection systems [8] and bioinformatics [9, 10].

One of the most popular offline algorithms, the FM-index, creates a Burrows-Wheeler transform plus some auxiliary data structures for rank support. While offline methods have a one-time overhead for creating an index, they typically have runtime advantages during the actual search. Common examples of offline algorithms are the suffix tree [11], suffix array [12] and most notably the FM-index. They all preprocess the text, e.g., a genome, to enable fast lookups. The FM-index provides the best theoretical runtime for single patterns of *O*(*m* + *occ*) to enumerate all hits, where *m* is the length of the pattern (query) and *occ* is the total number of hits found (assuming rank queries are constant time). For multiple patterns, the FM-index needs to be queried for each pattern separately. On the theoretical side, several works address indexed and bidirectional multi-pattern matching [13, 14]. For large texts and many queries, specialized large-scale homology and alignment tools like Lambda3 [15] and Diamond [16], which can process huge databases and queries, on the order of millions of sequences, exist. Their core are offline algorithms, like the FM-index used in Lambda, enabling efficient preprocessing of databases to maximize query efficiency. However, they are not suitable for the task of finding all exact matches, since they employ heuristics which are not fully sensitive.

Determining which algorithms perform best requires consideration of many factors, e.g., alphabet size, number and length of patterns, text length, hardware and implementation details. For some general guidelines, see Navarro & Raffinot [1] for different pattern-, and alphabet sizes. In proteomics, the number of patterns is on the order of tens to hundreds of thousand, making AC a prime choice. For exact matches, the Wu-Manber algorithm should be considered, since it is memory efficient and scales well with a large number of patterns. The multiple pattern matching becomes more challenging when wildcard support is required. A common task in bioinformatics, and proteomics in particular, is to find all exact and approximate matches of a set of peptide sequences in a proteome database, where peptides are infixes of proteins. Typical peptide lengths, as digested by trypsin and detectable by mass spectrometry, range from about 7 to 40 amino acids, while intact protein sequences in common proteomes vary greatly in length, typically from a few dozen to several thousand amino acids, averaging about 400 amino acids per protein. While our benchmarks use tryptic peptides for realism, Wild-AC itself is agnostic to how the patterns (peptides) were generated and is equally applicable to non-tryptic peptides, e.g., from other proteases or non-specific cleavage.

Proteomics databases (i.e., a collection of protein sequences) commonly contain ambiguous amino acids (AAAs; see Table 1 for an overview), which act like wildcards. As detailed below, the amino acid alphabet has only four such ambiguous codes (*B*, *J*, *X*, *Z*).

**Table 1:** IUPAC table of ambiguous amino acids (AAAs)

IUPAC Code	Amino Acid Names	One/Three-letter Codes
B	Aspartic acid or Asparagine	D (Asp), N (Asn)
Z	Glutamic acid or Glutamine	E (Glu), Q (Gln)
J	Leucine or Isoleucine	L (Leu), I (Ile)
X	Unknown or any amino acid	all 20 standard amino acids

See Table 2 for wildcard statistics in common databases. Peptides identified from experimental data in mass-spectrometry- based bottom-up proteomics commonly do not contain wildcards.

**Table 2:** Common proteomics databases with varying amounts of ambiguous amino acids (AAAs)

Database	No. of proteins	No. of tryptic peptides^a^	Fraction of sequences with AAAs [%]	B	Count of X	Z	J	No. of tryptic peptides^a^ with AAAs	FASTA size [MB]
Yeast	6067	694 023	0.00	0	0	0	0	0	4
Human	83 587	6 952 224	10.08	0	8425	0	0	12 421	39
UniProt All	573 661	47 195 693	0.42	276	7993	249	0	15 745	273

To determine the number of peptides in each protein, we perform an *in-silico* tryptic digest, i.e., cleaving the protein sequence after every Lysine (K) or Arginine (R), unless followed by Proline (P). We allow up to two missed cleavages, where trypsin fails to cut at an expected cleavage site. Consequently, peptides may contain up to two internal K or R residues that remain uncleaved.

^a^with up to two missed cleavages.

Most peptide search engines ignore any sequences with ambiguous amino acids (AAAs). However, once a peptide sequence is identified, all possible protein mappings (including to an AAA-containing region) of that sequence should be considered. Thus, a mapping from peptides to protein sequences with AAAs needs to be able to resolve ambiguities. A common application is the computation of protein sequence coverage or protein inference.

A related problem, where patterns can contain wildcards, but the text may not, has been solved by PatMaN [17]. However, this approach uses explicit enumeration of all combinations of representative characters for each wildcard occurring in a query pattern, which is feasible at the query level (patterns are short and few wildcard positions occur per pattern), but impractical at the text level (the text is long, so the number of wildcard positions, and thus combinations, can be very large). Similarly, there are also algorithms for multi-pattern matching with wildcards based on suffix arrays [18], but these only allow wildcards in the patterns, not the text. We reiterate that, throughout this work, wildcards are exclusively supported in the text (i.e., the protein database), never in the patterns (i.e., the peptides). We note that most FM-index-based search-scheme implementations (or move-index equivalents, which are index structures similar to the suffix array) do allow wildcards in the text as well, simply by trying all possible text characters at each wildcard position; Columba [19] is one such example in genomics, where our FM(SS) contestant (see Section 2) is essentially the proteomics equivalent.

The next sections are structured as follows: the Methods section introduces general notation as well as the Wild-AC algorithm itself, and motivates which competing tools were included in the benchmark. The results section compares Wild-AC with FM-index based approaches, a q-gram based approach, as well as the Wu- Manber algorithm on typical use cases in proteomics.

## Methods

### Notation and definitions

**Definition 1** An alphabet Σ is a finite set, whose elements are called characters (or letters). The size of Σ is denoted by *σ* = ∣Σ∣.

For example, the DNA alphabet is Σ_*DNA*_ = {*A, C, G, T* }, the amino acid (AA) alphabet is Σ_*AA*_ = {*A, Y, C, D, N, F, G, H, I, L, K, W, M, O, P, E, Q, R, S, T, U, V* }.

In proteomics, the letters *B, J, Z, X* encode ambiguous amino acids (AAAs), see Table 1, which act as wildcards with restrictions (in the sense that only certain characters from Σ_*AA*_ are a valid substitute). The wildcard alphabet in proteomics is thus Σ_*AAA*_ = {*B, J, Z, X*}, where *X* can be considered a full wildcard, also known as ‘don’t care’ character.

For proteomics, we define a wildcard representative mapping *R*∶ Σ_*AAA*_ → {*A*|*A* ⊆ Σ_*AA*_} such that for each wildcard symbol *z* ∈ Σ_*AAA*_, *R*(*z*) ⊆ Σ_*AA*_. In other words, each AAA is assigned a subset of the amino acid alphabet consisting of the characters it may represent, e.g., *R*(*B*) = {*D*, *N*}.

**Definition 2** A string is a finite sequence of characters, drawn from Σ.

**Definition 3** Let P a multi-set of patterns *P* = {*p*_1_*, p*_2_*, …, p*_*d*_}, where *d* = |*P*| and each pattern *p*_*i*_ consists of characters in the alphabet Σ.

Patterns must not be unique in *P*, i.e., identical patterns can occur multiple times in *P*.

In proteomics, the text T of length *n* = |*T*| is a string from the alphabet Σ_*P*_ = Σ_*AA*_ ∪ Σ_*AAA*_ and patterns are strings from the alphabet Σ_*AA*_.

We define *aaa*_*max*_ as the maximum number of ambiguous amino acids allowed per match, a parameter used to limit computational cost.

The problem is to find all occurrences of all the patterns of *P* with at most *aaa*_*max*_ ambiguities in the text *T*. Two characters match if (a) they are identical or (b) at the text character is a wildcard and the pattern character is in the reference mapping of that wildcard. Two equal-length strings match if and only if their i-th characters match for all *i*. Note that since patterns only contain unambiguous characters from Σ_*AA*_ (never wildcards, see above), a wildcard in the text can only ever be matched against a concrete amino acid via *R*(⋅), e.g., *B* against *D* or *N*; two wildcard symbols (e.g., *B* and *X*) never need to be matched directly against each other (although this is possible in theory using Wild-AC).

### Wild-AC algorithm

The input to the Wild-AC algorithm is a multi-set *P* of *d* patterns, *P* = {*p*_1_*, p*_2_*, …, p*_*d*_}, each pattern of varying length, a text *T* of length *n*, and a maximum number of allowed AAAs *aaa*_*max*_ per match.

Our Wild-AC algorithm adds wildcard support (in the text) on top of the Aho-Corasick automaton, a finite state automaton for multi-string pattern matching. The original algorithm is described in Aho&Corasick [2] in full detail. In short, for building the trie, the set of patterns *P* is converted into a rooted trie *G*, augmented with suffix links (also known as failure function), which allow for efficiently finding suffixes of partial matches in *P*. When traversing the trie by reading characters from the input text one by one, the active state either a) advances from a parent node to its child node if an edge for the current input symbol exists, or b) follows suffix links, until (a) becomes possible or the root is reached without a valid child. Each node in the trie maintains a list of patterns which end at this node. This list explicitly includes all the patterns found when following the chain of suffix links. Thus, each list can contain patterns which are shorter than the depth of the node. The Wild- AC algorithm uses the AC algorithm to create the trie. To enable wildcard support during the search phase, we modify the original AC algorithm as follows: if the next character *t*_*i*_ from the text *T* is not in the wildcard alphabet, the normal AC traversal rules are used. We call the currently visited (=active) node in *G* the primary node. Upon encountering a wildcard in the text, we enumerate all possible representatives *R*(*t*_*i*_) = *r*_1_*…r*_*k*_ and proceed as follows: For all representative characters *r*_1_*…r*_*k*_ we create a new active node, termed a scout. A scout traverses a parallel path in *G* spawned from the primary. The primary itself simply follows *t*_*i*_. If no wildcard was used in any of the patterns, this effectively moves the primary to the root of *G*. In other words, we explore *k* + 1 possible paths using *k* scouts and one primary (which explores the *t*_*i*_ character). Both a scout and the primary can spawn new scouts, if later on another wildcard in the text is encountered. Scouts which are spawned from a parent scout are called a sub-scout. Parent scouts follow the *t*_*i*_ character from the text, just as the primary does when encountering an AAA. Thus, it is easy to see that the number of scouts is exponential in the number of consecutive wildcards. In practice, this depends on the input text and is only problematic for wildcards with many representatives and especially so, if two or more wildcards are direct successors. To optimize the runtime, we introduce three mechanisms: first, the maximal number of wildcards *aaa*_*max*_ in a match. Every time a scout/sub-scout consumes an AAA, its *aaa*_*free*_ counter is decreased by one. Scouts are created from the Primary with *aaa*_*free*_ = *aaa*_*max*_ and sub-scouts inherit their *aaa*_*free*_ counter from the parent scout. Subsequent wildcards in the text will cause the scout to be abandoned once the counter is depleted. Second, we look ahead in the text and split the text at any consecutive occurrences of the *X* wildcard which are longer than the *aaa*_*max*_ into two text segments, where the tail of the first and the head of the second segment contain exactly *aaa*_*max*_*X*. Any additional *X*s in the middle are discarded (as they cannot be part of a match, since this match would have more than *aaa*_*max*_ wildcards). This only works if no pattern contains an *X* (which is usually the case in proteomics) and the shortest pattern is longer than *aaa*_*max*_. Third, and most importantly, we devise a stopping criterion for all scouts, which causes scouts to be abandoned (deleted) if they lose the first wildcard in their path from the root. This drastically limits the number of scouts. In detail, each scout tracks the number of characters prior to the first wildcard (termed representative) in its path from the root to its current position in *G*. Initially, the scout stores the depth of the node it was spawned from, after reading its first wildcard. Every time the scout follows a suffix link, this wildcard-free prefix is reduced by the difference in depth between two nodes connected by a suffix link. Should the counter reach zero, the scout’s path has lost the representative, and thus the scout is deleted. Sub-scouts simply inherit their wildcard-free prefix from their parent scout as is. In other words, if a (sub-)scout’s path does not contain its representative anymore, any match would also be found by the primary, since the primary has read the same characters from the text after the scout’s representative was encountered. One last component of the Wild-AC algorithm is filtering of reported hits, to ensure each match is reported exactly once by the Wild- AC algorithm. In combination with the scout termination described above, this is required to avoid the need for post-hoc deduplication of reported hits. The primary reports all hits associated with its currently active state, no matter their length. When a scout has consumed a new text character and reached a new node in *G* without being terminated, it will only report patterns from its hit list which contain the scout’s representative, i.e. short hits may not be reported. In other words, the length of the hit must be larger than the scout’s depth minus its wild-card-free prefix.

The core algorithm’s pseudo code is shown in Algorithm 1. Note that in principle, the algorithm can also be used to find hits with mismatches (MMs). This causes an increase in runtime on the order of the alphabet size for every mismatch and is usually not favorable, although the implementation supports this. In short, if MMs are allowed, all members of the alphabet not explored by either wildcard- scouts or the primary are explored by MM scouts. Destruction of MM spawns and reporting of hits follows the same rules as for wildcard scouts. The pseudocode in Algorithm 1 only considers the case of wildcards for the sake of clarity.

**Fig. 1:**
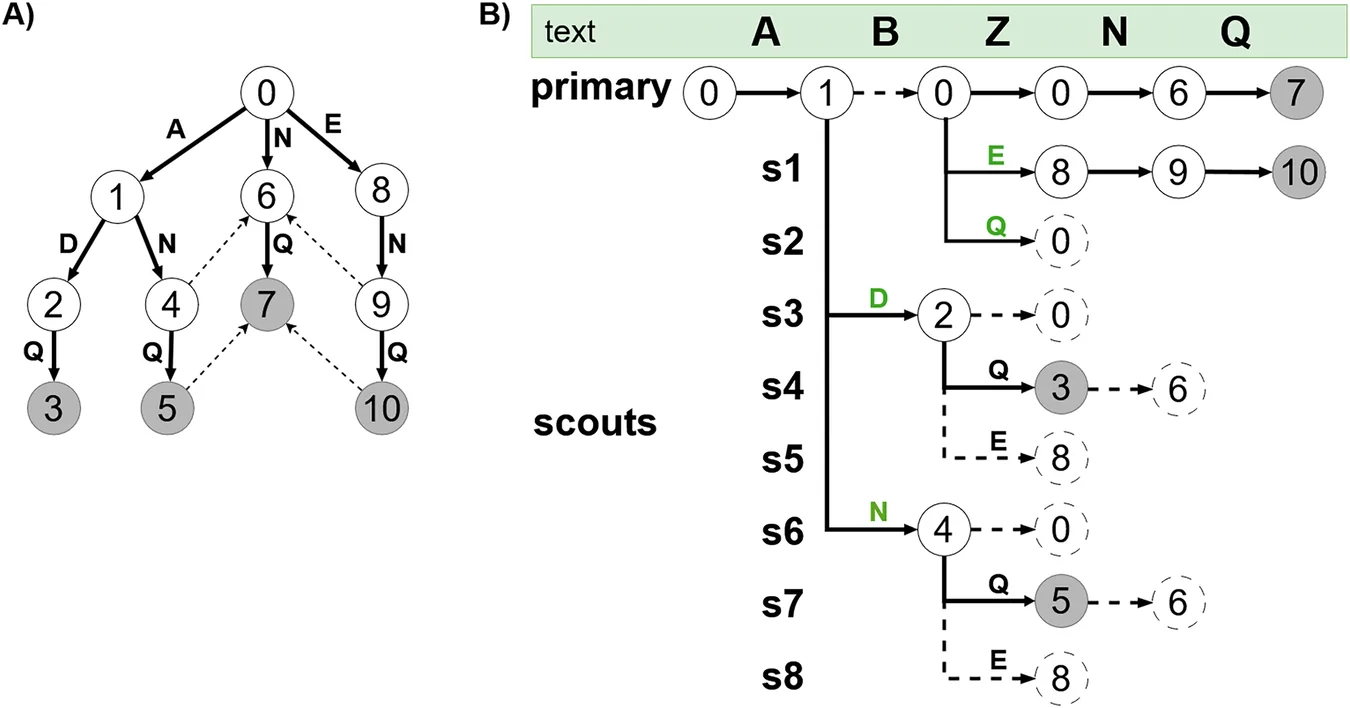
A Pattern matching machine for the patterns: {*ADQ, ANQ, NQ, ENQ*}. Nodes are numbered in order of trie creation. Edges are labeled with the character which allows transition from parent to child node. Output nodes are shown in grey. Every node, except the root, has a suffix (=failure) link, which are shown as dashed edges. For brevity, a suffix link is visually omitted if it points to the root node. **B** Sequence of state transitions of the primary and scouts (s1 - s8) for the text *ABZNQ*, where *B* = {*D, N* } and *Z* = {*E, Q*}. Representative characters for each scout are colored in green. Output nodes are shown in grey. Scouts spawning from scouts inherit the representative character of their parents. Edges are solid if reading from the input just requires following a direct edge to the child node; dashed edges require following one or more suffix links to match the character, and may therefore chain through multiple nodes, including an intermediate hop via the root node (e.g., node 3 has no outgoing *N* edge and its (visually omitted) suffix link leads to the root, whose *N* edge leads to node 6; the dashed edge 3 ⇢ 6 for scout s4 depicts this combined traversal, not a single suffix link). Nodes are drawn with a dashed outline if a scout is terminated there due to loss of the representative (dashed outlines are unrelated to the dashed edges)

By construction, no active node (scout or primary) follows the same path, i.e., are in the same state. This design ensures that hits are never reported twice (i.e., either the primary or a scout reports a hit). A compact example is shown in Fig. 1 for the patterns {*ADQ, ANQ, NQ, ENQ*} and text *ABZNQ* which contains *B* and *Z* as wildcards. In total five hits can be found: *ADQ*, *ANQ*, *NQ*, *ENQ* and *NQ* at text positions 1, 1, 2, 3 and 4 respectively. Whenever a wildcard is encountered in the text, e.g., *B* in position 2, the algorithm will spawn scouts for all representatives of that AAA. Scouts continue reading from the input until they lose their representative AA. This happens by following suffix links in the trie, which effectively cause a scout to lose a certain prefix in its path. For example, when exploring the path *ADE*, which follows the sequence of nodes 0, 1, 2, 8, a scout (s3) is spawned at node 1 when reading *D*, representing *B* from the text. Immediately afterwards a *Z* is read from the input, thus the scout branches off sub-scouts (s4, s5) to explore the representatives of *Z*. The sub-scout s5 moves from node 2 at depth 2 to node 8 at depth 1. Thus, the scout loses a prefix of length two (the *AD*), which includes its representative (the *D*) and is therefore terminated. The mechanism to avoid duplication of hits can also be observed in Fig. 1: When scout s7 reaches node 5, it reports the immediate hit *ANQ* at this position, as well as the suffix hit *NQ* (at node 7), since its representative (the *N*) is contained in both hits. On the other hand, scout s1 reports *ENQ* when reaching node 10, but does not report the suffix hit *NQ*, since it is too short and does not contain its representative (the *E*).

#### Algorithm 1

Pseudocode of the Wild-AC search algorithm. The failure function ‘f()‘ and the goto function ‘g()‘ are the Aho-Corasick equivalents.

1: **procedure** Wild-AC search (graph G, string text, int *aaa*_*max*_)

2: *node* ← *root*(*G*)

3: *scouts* ← {}

4: **for** c in text **do**

5: *node* ← *traverse*(*G, node, c*) ▷ walk Primary in G

6: *reportHits*(*node*)

7: **for** S in scouts **do **▷ process existing scouts

8: **if** c in {*B, Z, J, X*} and *S.aaa*_*free*_ > 0 **then**

9: **for** aaa in ambiguitiesOf(c) **do **▷ create more scouts?

10: *subscout* ← *createScout*(*S.node, S.prefix left,*

*S.aaa*_*free*_ − 1)

11: **if** !traverseScout(subscout, G, aaa) **then**

12: continue ▷ this subscout lost its AAA prefix

13: **end if**

14: insert(scouts, subscout)

15: reportHitsScout(subscout)

16: **end for**

17: **end if**

18:

19: **if** !traverseScout(S, G, c) **then **▷ traverse S itself

20: delete S from scouts

21: **else**

22: reportHitsScout(S)

23: **end if**

24: **end for**

25:

26: ▷ create new scouts from primary

27: **if** c in *{*B,Z,J,X*}* and *aaa*_*max*_ > 0 **then**

28: **for** aa in ambiguitiesOf(c) **do **▷ e.g. *{*D, N*}* for c = B

29: *node*_*s*_ ← *traverse*(*G, node, aa*)

30: **if***node*_*s*_ ≠ *root*(*G*) **then **▷ AA was matched

31: *S* ← *createScout*(*node*_*s*_*, depth*(*node*_*s*_)*, aaa*_*max*_ − 1)

32: insert(scouts, S)

33: reportHitsScout(S)

34: **end if**

35: **end for**

36: **end if**

37: **end for**

38: **end procedure**

39:

40: **procedure** traverse(Graph G, Node s, Char c)

41: **while** g(s, c) = fail **do**

42: s ← f(s)

43: **end while**

44: *s* ← *g*(*s, c*)

45: return *s*

46: **end procedure**

47:

48: **procedure** traverseScout(Scout S, Graph G, Char c)

49: *old depth* ← *depth*(*S.node*)

50: *S.node* ← *traverse*(*G, S.node, c*)

51: *S.prefix left* -= *old depth* − *depth*(*S.node*) + 1

52: return *S.prefix left* > 0 ▷ retain first AAA in path from root?

53: **end procedure**

54:

55: **procedure** reportHitsScout(scout)

56: *node* ← *scout.node*

57: *min patt length* ← *depths*(*node*) − *scout.prefix left*

58: **for** hit in hits(node) **do**

59: **if ***hit.length* > *min patt length ***then**

60: print hit ▷ hit contains the representative

61: **end if**

62: **end for**

63: **end procedure**

### Benchmarking procedure

We will compare our Wild-AC algorithm with FM-index based approaches on typical use cases in proteomics, where a set of patterns (peptides), typically with an average length of 18 amino acids, is matched to a proteome, ranging from a few megabytes to hundreds of megabytes in size, while allowing for restricted wildcards (here: ambiguous amino acids).

#### Input protein databases

We choose three databases from UniProt, as listed in Table 2: Yeast (4 MB), Human (39 MB) and UniProt-All (273 MB), which are commonly used in proteomics. We note that the Human database is similar to Mouse in terms of structure and size (28 MB), and we randomly decided to use the Human database to reduce redundancy in the results. Additionally, we created a masked copy of each database, in which every residue is replaced by *X* with a probability of 5%, while the peptide sets remain those sampled from the unmasked database. We mask with *X*, since it has the highest branching factor of all AAAs, representing all 20 standard amino acids (see Table 1), whereas *B*, *Z* and *J* have two representatives each.

#### Input peptide sequences

We use four peptide input sets of varying size: 1000, 10 000, 100 000 and 500 000 peptide sequences. These sizes reflect the possible bandwidth of typical bottom-up shotgun experiments in proteomics. Peptide sequences are randomly sampled from each input database after a complete tryptic in-silico digest with up to two missed cleavages. Any ambiguous amino acids in the sampled peptides are resolved randomly to one of its representatives (e.g., a *B* may be resolved to *N*). Once sampled, the peptide sets stay fixed and are used as input to all algorithms. Due to sequence similarities in the database, the peptide sets may contain duplicate sequences. This is also typical in proteomics, due to oversampling and post-translational modifications, which results in the same peptide sequence being identified multiple times in a single experiment.

#### Algorithms

We include the following contestants to compete against the Wild- AC algorithm:

##### PeptideMapper

The PeptideMapper [20] software, which builds upon an FM-index, was developed for mapping peptides to protein databases with ambiguities. Initial benchmarks showed that PeptideMapper (version 5.1.9) is slower than other FM-index implementations. Additionally, PeptideMapper’s parametrization does not allow explicitly setting the number of AAAs or mismatches, only relative frequencies, which complicates comparison of results. Thus, to maximize competitiveness and comparability, we use the highly optimized FM-index Collection (see below).

##### FM-index collection

The FM-index Collection [21], written in C++, is highly optimized for speed and offers a broad range of FM-index flavors. It supports the standard backtracking algorithm, FM(BT), which explores all possible wildcard paths using brute force (identical to PeptideMapper’s implementation), as well as search schemes [22], FM(SS), which partitions a query into substrings and allows efficient searching with given mismatch and wildcard bounds. The implementation offers specific optimizations for ambiguous amino acids. The runtime measurements only include the search phase, since the index is created prior to the benchmark and reused for multiple searches. The index creation time is reported separately in Supplementary Table A.4.

##### Wu-Manber

Wu-Manber (WM) [5] is often cited as competitor of Aho-Corasick and is widely used. We use an efficient C++ implementation of the original Wu-Manber algorithm to showcase its performance for the exact search case, i.e., wildcards are not supported.

##### ProteoMapper

ProteoMapper [23] is written in Perl and advertises fast mapping from peptides to proteins, including support for fuzzy searching with mismatches (but not wildcards). It supports sequence variants via PEFF files and preprocesses the text by creating an index akin to a Q-Gram index for a fast segmented lookup. Notably, ProteoMapper excludes peptides which contain any of the amino acids {*J, U, Z, B, O, X*} from the search. Consequently, hits in the text where any of these amino acids occur are missed. Luckily, our peptide sets only contain a handful of peptides with the amino acids pyrrolysine (*O*) and selenocysteine (*U*) and, as previously described, no AAAs.

#### Wall time benchmark

We measure wall clock time for the entire application including loading of input data (index or FASTA file), since this reflects the user experience. The creation of an index (if any) is not part of the benchmark. Benchmarks were run on a server with an AMD EPYC 7702 P 64-Core Processor and 1 TB of RAM using Debian 12. All C++ algorithms were compiled with -march = native and -O3.

#### Memory consumption benchmark

To assess each algorithm’s memory requirement we record the maximum resident set size (RSS), i.e., peak memory consumption. The memory footprint includes the input data. We note that our Wild-AC implementation is partially optimized for memory consumption by making use of a breadth-first trie datastructure with bit fields for child indexing, which reduces the space requirements for the tree itself to about 16 bytes per node (without accounting for storage on which patterns end at each node).

## Results

We first evaluate correctness of the implementations (summarized by the number of hits). Our Wild-AC implementation is additionally verified using a dedicated unit test suite covering the trie construction and scout-based traversal logic (see 6.3). When working on weaker hardware, the memory requirements potentially become a concern, thus we explore the memory footprint as well and finally benchmark algorithm runtime on a variety of input sets and parameter settings.

### Correctness and number of hits

With the exception of ProteoMapper, all other algorithms, namely FM-index, Wild-AC and Wu-Manber report identical number of hits. Only ProteoMapper disagrees on the number of hits, in particular as the number of patterns increases. This is due to internal deduplication of peptide query sequences: if a peptide occurs multiple times in the input, results are only reported once by ProteoMapper. Another, albeit smaller, contribution is that ProteoMapper excludes peptides containing selenocysteine or pyrrolysine. Lastly, ProteoMapper does not support AAAs, but only mismatches, which increases the number of hits considerably. Supplementary Table A.5 lists the number of hits for each combination of algorithm, protein database and peptide input set. Due to the relatively low number of wildcards in proteomic databases (see Table 2), the number of hits increases only mildly when allowing for wildcards. The yeast database does not contain any ambiguous amino acids, thus the number of hits is identical for exact and wildcard search, as are the runtime measurements (see top row of Fig. 3a; except for FM(SS), see the Runtime subsection for details). The human database yields a very small increase in the number of hits for a single wildcard (≈ 0.1%), yet does not increase further for two wildcards. The UniProt-All database shows a more pronounced increase with up to ≈ 11% more hits for five wildcards.

### Runtime

Table 3 shows the theoretical worst-case runtime we can expect for each algorithm. By design, Aho-Corasick based approaches are favorable, when the number of patterns is large, and the input text is small. On the other hand, index-based approaches like the FM- index, usually work well on large input texts, long patterns, and small alphabets.

**Table 3:** Worst-case runtime for preprocessing, index creation and exact/wildcard search with *d* (number of patterns), *m* (average pattern length), *occ* (number of hits), *n* (text length), *σ* (alphabet size), *w* (number of allowed wildcards per hit), *q* (q-gram length of the ProteoMapper index, *q* = 5) and *B* (block length of the Wu-Manber *B*-gram tables)

Algorithm	Index creation	Pattern pre- processing	Search time
Wild-AC	–	*O*(*dmσ*)	*O*(*n****σ***^***w***^+ occ)
FM(BT)	*O*(*n* log *σ*)	–	*O*(*dm****σ***^***w***^ log *σ* + occ)
FM(SS)	*O*(*n* log *σ*)	–	*O*(**(*w*+1)***dm****σ***^***w***^ log *σ* + occ)
ProteoMapper	*O*(*n* + *σ*^q^)	–	*O*(*dmn* + occ)
Wu-Manber	–	*O*(*dm* + *σ*^*B*^)	*O*(*nmd* + occ)

The search time column states the wildcard search, where the **bold** subterms are the wildcard-specific factors; they degenerate to 1 for exact search, i.e., dropping them yields the exact-search runtime. ProteoMapper and Wu-Manber support exact search only. Absolute wall-clock numbers for pattern preprocessing (Wild-AC) and index creation (FM-Index) are provided in Supplementary Tables A.6 and A.4, respectively.

Wild-AC’s pattern preprocessing (i.e., trie construction) is a pure function of the number and average length of the input peptides: it does not depend on the target protein database or the number of allowed ambiguous amino acids (*aaa*_*max*_). Measured absolute preprocessing times range from about 2 ms (1000 peptides) to about 1.4 s (500 000 peptides), i.e., roughly 2 µs per peptide; see Supplementary Table A.6 for details. Conversely, FM-index construction depends only on the protein database being indexed, independent of any peptide set; see Supplementary Table A.4.

The benchmarks align with the theoretical scaling behavior in the number of patterns *d* vs. text length *n*. However, the asymptotic exponents in Table 3 should not be used to predict the actual quotient of measured wall-clock times, since both algorithms are governed by different, constant factors that big-O notation intentionally hides. For example, Wild-AC’s AC-trie traversal performs one cache- friendly, sequential array/bit-field lookup per text character in the common (wildcard-free) case, whereas each FM-index rank query touches several, less predictable memory locations in its auxiliary rank-support structures.

**Fig. 2:**
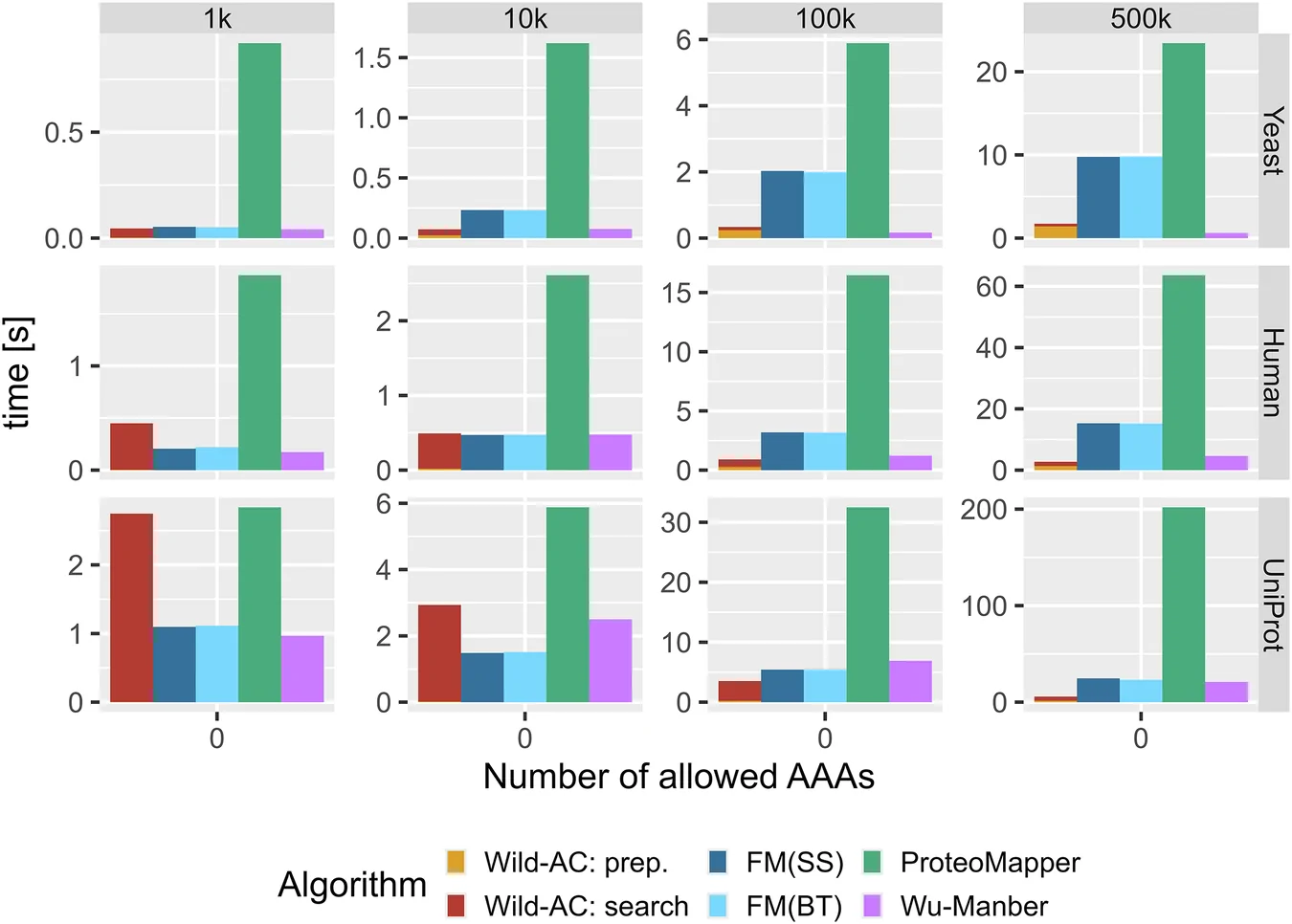
Exact search time benchmark. The number of patterns increases across columns, and database size increases down rows. The runtime for Wild-AC is split into preprocessing and actual search (yellow/red bars). Note that the y-axes are scaled per panel

For the exact search, which is supported by all algorithms, the FM-index beats Wild-AC when the database size is larger (Human or UniProt-All) and the number of patterns is below 10 000 – see Fig. 2. When the number of patterns increases (≥100k), Wild-AC clearly wins, being up to 6 times faster than the FM-index (see 100k/500k, Yeast). Notably, for small databases (Yeast) Wu-Manber outperforms every other algorithm (see top row Fig. 2) for the exact search. ProteoMapper is the slowest in any combination of database and pattern set.

When handling wildcard patterns, support is limited to FM-index variants that incorporate search schemes (SS), backtracking (BT), and the Wild-AC approach. Wu-Manber and ProteoMapper do not support amino acid wildcards.

**Fig. 3:**
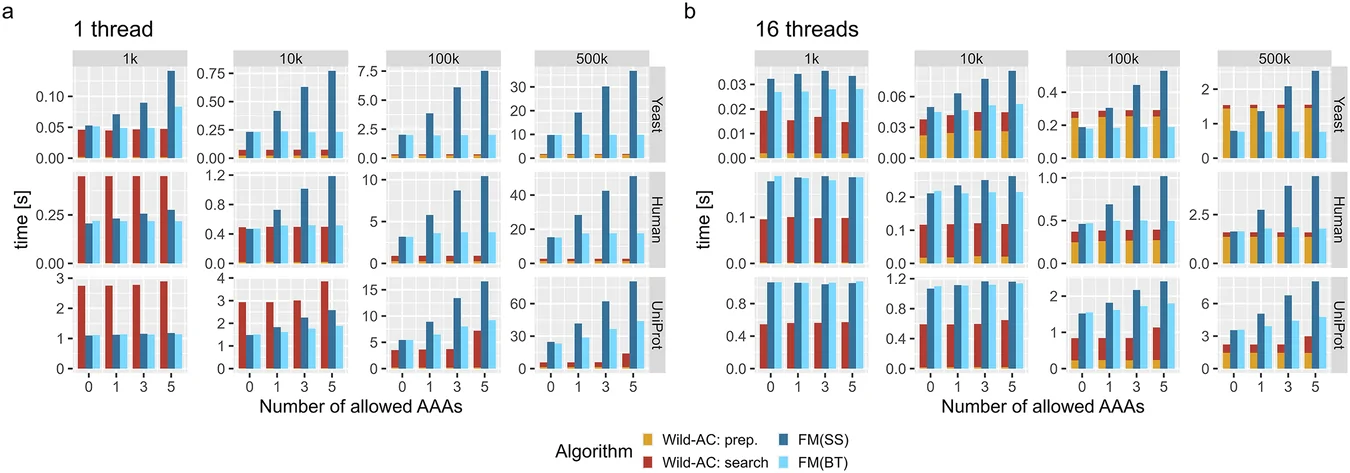
Wildcard search time benchmark for different number of ambiguous amino acids, using **(a)** a single thread and **(b)** 16 threads. The number of patterns increases across columns, and database size increases down rows. The runtime for Wild-AC is split into preprocessing and actual search (yellow/red bars). Note that the y-axes are scaled per panel

Similar to the exact search, the FM-index variants perform well for smaller number of peptide patterns and larger databases - see Fig. 3a. Notably, for a large number of patterns and smaller databases (such as Yeast, 500k peptides), Wild-AC spends 80% of its runtime in the preprocessing phase, but still outperforms the FM-index variants by a factor of 5.5 (FM(BT)) and 21.2 (FM(SS)).

Interestingly, the performance of SS and BT diverges for larger number of AAAs. The optimized search scheme implementation requires more time with increasing number of wildcards. On the unmasked databases, the naive backtracking is always faster. We suspect the search-scheme partitioning overhead (splitting the query and managing multiple index ranges) does not pay off when the number of allowed wildcards is large compared to the query length. Single-threaded, the Wild-AC algorithm beats any FM-index for moderate to large peptide input sets (≥100k), irrespective of database size.

Finally, we measured multi-threading performance for Wild-AC and FM-index variants. The Wild-AC implementation is parallelized over proteins in the input database. Conversely, the FM-index is parallelized across peptide sequences. At 16 threads the total runtime is reduced by up to 6.4x for Wild-AC and 12.8x for FM(BT). Interestingly, with increasing database size and decreasing number of patterns, Wild-AC scalability increases. This is due to the constant overhead of the currently single-threaded trie creation in Wild-AC. Unsurprisingly, FM-index parallelization scales better for smaller databases and increasing number of patterns. See Fig. 3b for details. This causes Wild-AC to win across all peptide input set sizes, for the Human and UniProt-All databases. The winner for small databases (Yeast) depends on the peptide input set size.

**Fig. 4:**
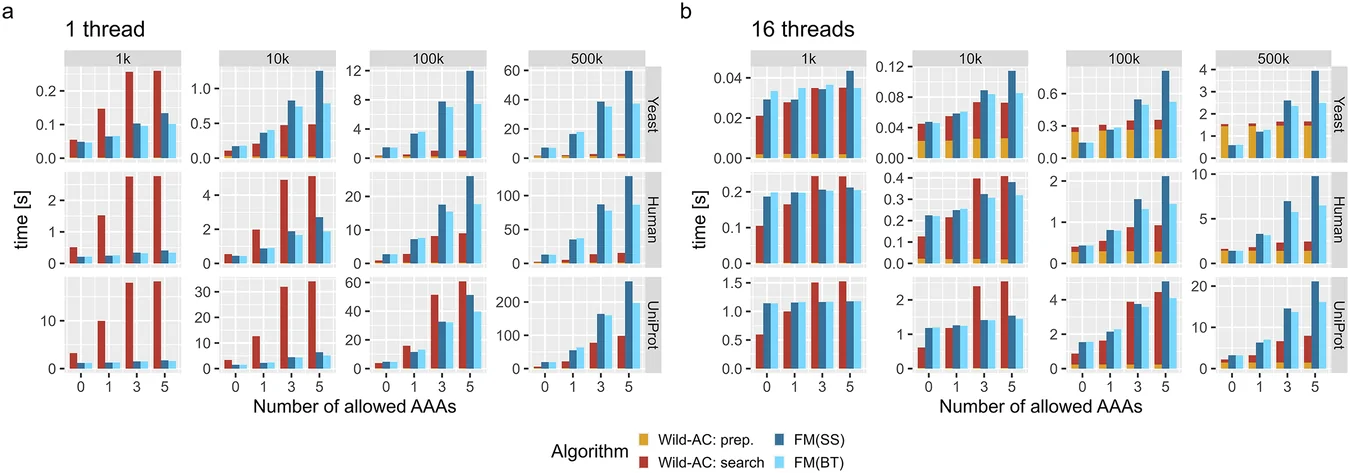
Wildcard search time benchmark on the 5%-masked databases, using (a) a single thread and (b) 16 threads. Layout as in Fig. 3

Finally, we explored applications with a higher wildcard rate than proteomes offer (see Table 2), using the 5%-masked databases – see Fig. 4. The ranking is unchanged in principle, but the break-even point moves towards larger peptide sets: at *aaa*_*max*_ = 5 and a single thread, Wild-AC wins from 10 000 peptides onwards for Yeast, from 100 000 for Human and from 500 000 for UniProt-All (by a factor of 12.5, 5.6 and 2.0 over FM(BT), respectively), while the FM-index is up to 11 times faster for the smallest peptide sets. At 16 threads, the masking shifts the balance further in Wild-AC’s favour for large peptide sets, since the additional wildcard work is parallelized while trie creation is currently not: with 500 000 peptides, trie creation drops from 49 to 18% of the runtime for UniProt-All, while Wild- AC’s lead over FM(BT) more than doubles for Human (to 2.6×). The gap between FM(SS) and FM(BT) still widens with the number of wildcards on the masked databases, but far more slowly than on the ^unmasked ones (single-threaded, 1.4× vs. 2.1× at *aaa*^*max* ^=^ ^5).^

### Memory consumption

**Fig. 5:**
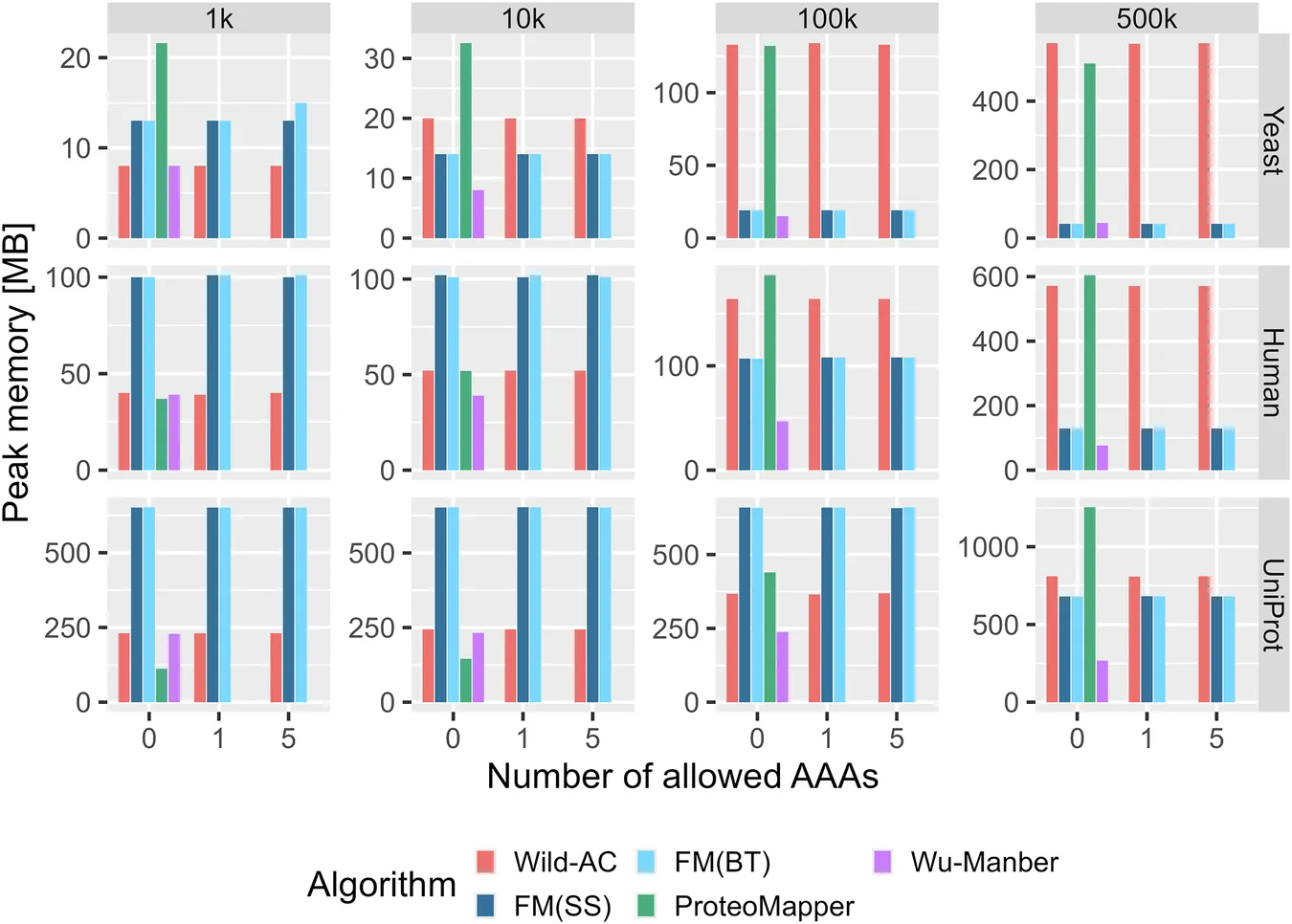
Peak memory consumption (RSS) of each algorithm for 1000, 10 000, 100 000 and 500 000 peptide sequences for Yeast, Human and UniProt-All databases

Peak memory usage across all algorithms remained below 1.3 GB in the search phase, even for the largest input data (273 MB UniProt-All database; 500k peptide sequences), see Fig. 5.

For Wild-AC and Wu-Manber, memory consumption increases with larger number of patterns, whereas index-based algorithms require more memory when the text size (and thus the index) grows. For large datasets (UniProt-All database with 500k peptides) the FM- index, Wild-AC and ProteoMapper have very similar requirements of around 750 MB of RAM when using a single thread, and only a few MB more per thread in multi-threaded runs (data not shown). Wu- Manber is always one of the most space efficient algorithms across all input sizes.

Index-based algorithms may use up to 2.5 GB of RAM during index construction. See Supplementary Figure B.6 for details.

## Discussion

Wild-AC is an extension of the well-known Aho-Corasick (AC) algorithm, operating on the same trie data structure but enhanced with specialized traversal rules to support wildcards in the text. Despite its theoretical worst-case runtime of *O*(*nσ*^*w*^) when the number of allowed wildcards *w* increases, Wild-AC performs exceptionally well in real-world proteomics scenarios. In practice, wildcards are rare and typically limited to *X* (unknown amino acid), with *B*, *Z*, and *J* occurring at low frequencies (see Table 2). This also causes the runtime to only mildly increase with the number of wildcards (for both Wild-AC and FM-index variants, compare Table 3), since a single peptide sequence rarely spans multiple wildcards (with Uniprot-All being the most extreme case). This makes Wild-AC highly practical for current workflows. When wildcard prevalence increases to 5%, as in our synthetic benchmark, the single-threaded runtime for Wild-AC increases by a factor between 1.7 and 10.3 (at *aaa*_*max*_ = 5), and that of FM(BT) only between 1.2 and 4.9, since Wild-AC spawns scouts at every wildcard in the text, whereas the FM-index pays per query character; accordingly, Wild-AC’s runtime increases 1.7× for Yeast with 500 000 peptides, where the total pattern length exceeds the database, but stays near 7× for UniProt-All, where the database exceeds the total pattern length by more than an order of magnitude. The algorithm’s performance advantage over the FM-index - especially for large pattern sets (≥100k peptides) - stems from the AC trie’s ability to scale well with pattern count. When using multi-threading, Wild-AC outperforms the FM-index even for smaller peptide sets, provided the database is large enough. This is likely due to CPU cache effects: the FM-index scales well only when the index fits in cache, while the AC trie’s preprocessing overhead is offset by better scalability with increasing database size (i.e., number of proteins to search through). A key limitation of Wild-AC is its exponential growth in the number of scout paths under consecutive wildcards. However, this is mitigated by three mechanisms: (1) a maximum allowed number of wildcards per match (*aaa*_*max*_), (2) a look-ahead optimization that discards consecutive *X* characters beyond *aaa*_*max*_, and (3) a scout termination rule that deletes paths losing their representative amino acid. These ensure computational feasibility in real-world settings.

The implementation is optimized for C++ and supports multi- threading, with a didactic version available on GitHub that provides full traceability for debugging and educational use. Future work will explore multi-threaded trie creation and compressed AC trie variants (e.g., double arrays and their compressed forms [24]) to further optimize memory and speed on modern hardware.

Finally, the ProteoMapper tool, which relies on q-grams and is implemented in Perl, was not competitive in our benchmarks- likely due to both algorithmic inefficiency and language-level overhead. This highlights the importance of low-level optimization in high-throughput bioinformatics tools.

## Conclusion

The Wild-AC algorithm addresses a critical gap in multi-pattern string matching: efficient wildcard support in the text, not just the patterns. While FM-index-based approaches are highly optimized for large databases and exact or mismatch-based searches, they require costly preprocessing and suffer from scalability issues when the number of patterns increases. In contrast, Wild-AC avoids index construction entirely, making it especially suitable for dynamic or rapidly changing proteome databases where frequent re-indexing is impractical, while also reducing workflow complexity. Wild-AC is a fast, index-free, multi-pattern string matching algorithm that supports wildcards in the text, specifically designed for proteomics applications. It outperforms the FM-index in wildcard search and matches or exceeds Wu-Manber in exact search for large-scale peptide mapping tasks. By eliminating the need for preprocessing of the text, Wild-AC enables rapid, scalable analysis of large proteome databases at full sensitivity. Its open-source implementation, multi-threading support, and integration into OpenMS [25] ensure broad accessibility and reproducibility. The didactic version of Wild-AC (available on GitHub) provides full traceability for debugging and educational use. 

## Supplementary information

Supplementary Material 1

## Data Availability

We provide a standalone C++ implementation of Wild-AC (v1.0) at https://github.com/Wild-AC/Wild-AC in two flavors. Oneflavor (performance) is tuned for runtime and was used in the benchmarks. The other flavor (didactic) focuses on a straightforward implementation and provides verbose command line output, which allows tracing the graph traversal. Additionally, Wild-AC is available in the OpenMS software library [25] as part of the PeptideIndexer TOPP tool. Our C++ implementation of Wu-Manber (v1.0) is heavily based on code by Ray Burkholder and available at https://github.com/Wild-AC/WuManber C++ implementation of the FM-index by Gottlieb, which was used in our benchmarks is available at https://github.com/Wild-AC/FM-Index (v1.6), last updated 3/3/2023, was downloaded from http://tppms.org/pm/, with a mirror at https://github.com/Wild-AC/ProteoMapper (v5.1.9) was obtained from the Compomics suite at http://genesis.ugent.be/maven2/com/compomics/utilities/5.1.9/utilities-5.1.9.zip proteome data for yeast (UPID: UP000002311), human(UPID: UP000005640), mouse (UPID: UP000000589) and UniProt-All were obtained in FASTA format from UniProt[26] in July 2025 and are available at https://github.com/Wild-AC/data_and_scripts, along with scripts to re-run the benchmarks.

## Declarations

### Authors’ information

CB is a post-doctoral researcher with a focus on efficient algorithms and quality control in proteomics. He currently leads the Proteomics Section at the Bioinformatics Solutions Center, Freie Universität Berlin.
