# Supplementary material for: Wild-AC: A fast, index-free multi-pattern string matching algorithm with wildcard support for proteomics
Source: BMC Bioinformatics. 2026 Oct 9;27:234. doi: 10.1186/s12859-026-06686-8 (PMC13659980; doi:10.1186/s12859-026-06686-8)
Supplement: Supplementary file 1 [file 12859_2026_6686_MOESM1_ESM.docx]

# Supplementary Tables

**Table A.4** FM-index construction (index creation) wall-clock time across the proteome databases (Human, Yeast, UniProt-All; see Table 2), comparing 1 thread against 16 threads. Unlike Wild-AC’s pattern preprocessing (Table A.6), which depends only on the patterns and is independent of the target database, FM-index construction depends only on the protein database (text) being indexed, independent of any peptide/pattern set.

| Protein DB | 1 thread | 16 threads | Speedup (1 vs. 16) |
| --- | --- | --- | --- |
| Yeast | 0*.*48 s | 0*.*45 s | 1.07× |
| Human | 5*.*16 s | 3*.*79 s | 1.36× |
| UniProt-All | 43*.*08 s | 27*.*80 s | 1.55× |

**Table A.5** Number of hits for each combination of peptide input set and proteome database (see Table 2). The left part reports the number of hits for exact search (*aaamax* = 0). ProteoMapper reports hits for duplicate input peptides only once. The number of hits may therefore be lower for ProteoMapper. All other algorithms (Wild-AC, FM(SS), FM(BT), Wu-Manber) report hits for each individual peptide and agree on the number of hits in each configuration. The right part reports the relative increase in the number of hits (in %, relative to ‘All others’ at *aaamax* = 0) when up to *aaamax* ambiguous amino acids are allowed per match (Wild-AC, FM(SS), FM(BT); Wu-Manber and ProteoMapper do not support AAAs). For Yeast, allowing AAAs yields no additional hits; for Human, all additional hits require only a single AAA.

Hits (*aaa_max_* = 0)	Additional hits [%] for *aaa_max_*

| Number of Peptides | Protein DB | ProteoMapper | All others |  | 1 | 2 | 3 | 4 | 5 |
| --- | --- | --- | --- | --- | --- | --- | --- | --- | --- |
| 1000 | Yeast | 1015 | 1015 |  | 0.000 | 0.000 | 0.000 | 0.000 | 0.000 |
|  | Human | 3589 | 3589 |  | 0.167 | 0.167 | 0.167 | 0.167 | 0.167 |
|  | UniProt-All | 32 587 | 32 587 |  | 0.003 | 0.009 | 0.031 | 0.304 | 2.955 |
| 10 000 | Yeast | 22 489 | 22 703 |  | 0.000 | 0.000 | 0.000 | 0.000 | 0.000 |
|  | Human | 60 354 | 60 456 |  | 0.093 | 0.093 | 0.093 | 0.093 | 0.093 |
|  | UniProt-All | 121 900 | 123 587 |  | 0.138 | 0.196 | 0.270 | 0.908 | 8.357 |
| 100 000 | Yeast | 127 293 | 208 651 |  | 0.000 | 0.000 | 0.000 | 0.000 | 0.000 |
|  | Human | 613 137 | 710 567 |  | 0.062 | 0.062 | 0.062 | 0.062 | 0.062 |
|  | UniProt-All | 819 042 | 903 691 |  | 0.064 | 0.095 | 0.194 | 0.982 | 10.848 |
| 500 000 | Yeast | 516 897 | 1 053 280 |  | 0.000 | 0.000 | 0.000 | 0.000 | 0.000 |
|  | Human | 2 161 063 | 3 500 623 |  | 0.065 | 0.065 | 0.065 | 0.065 | 0.065 |
|  | UniProt-All | 3 360 144 | 4 726 084 |  | 0.057 | 0.088 | 0.181 | 0.939 | 10.568 |

**Table A.6** Wild-AC pattern (trie) preprocessing time for different peptide sets across the proteome databases (Human, Yeast, UniProt-All; see Table 2) where the peptides were randomly sampled from. Preprocessing time depends only on the number and average length of the input peptides. It does not depend on the number of allowed ambiguous amino acids (*aaa_max_*), or proteome database size, or the number of threads (since trie

construction is currently single-threaded). The rightmost column reports the average preprocessing throughput per peptide (in µs, averaged across the three databases shown).

|  | | Runtime [s] for each source database |  | |
| --- | --- | --- | --- | --- |
| Number of Peptides | Human | Yeast | UniProt-All | Throughput [µs/peptide] |
| 1000 | 0.0020 ± 0.0002 | 0.0020 ± 0.0002 | 0.0017 ± 0.0000 | 1.900 |
| 10 000 | 0.0196 ± 0.0016 | 0.0234 ± 0.0009 | 0.0165 ± 0.0002 | 1.983 |
| 100 000 | 0.2653 ± 0.0051 | 0.2406 ± 0.0019 | 0.2251 ± 0.0031 | 2.437 |
| 500 000 | 1.3466 ± 0.0095 | 1.4092 ± 0.0081 | 1.4199 ± 0.0109 | 2.784 |

# Supplementary Figures

**Figure B.6** Peak memory consumption (RSS) of each algorithm for 1000, 10 000, 100 000 and 500 000 peptide sequences for Yeast, Human and UniProt-All databases, including index creation for FM-index and ProteoMapper.

# Console output for didactic Wild-AC

Using the didactic version of Wild-AC (see <https://github.com/Wild-AC/Wild-AC/tree/main/didactic>) on the input of Figure 1 (text: ABZNQ, patterns: ADQ, ANQ, NQ, ENQ) with up to two allowed AAAs:

$ WildAC 2 ABZNQ ADQ ANQ NQ ENQ

produces the following output:

Loaded protein ABZNQ and 4 peptides

Build trie with 11 nodes and 4 needles. Trie has 11 nodes

Primary following edge A from node 0 to node 1

\# Created scout following edge D from node 1 to node 2 # Created scout following edge N from node 1 to node 4 Primary following edge B from node 1 to node 0

\## Creating sub-scouts from current scout at node 2... Sub-Scout created at node 2, exploring edge E ...

--> sub-scout died at node 8 (due to prefix loss of 2) Sub-Scout created at node 2, exploring edge Q ...

!!! Hit found (Scout): ADQ at text-position 0

--> sub-scout reached node 3 (will be put in scout queue) ## End sub-scout creation

--> Scout died following edge Z from node 2 to node 0

(due to prefix loss of 3)

\## Creating sub-scouts from current scout at node 4... Sub-Scout created at node 4, exploring edge E ...

--> sub-scout died at node 8 (due to prefix loss of 2) Sub-Scout created at node 4, exploring edge Q ...

!!! Hit found (Scout): ANQ at text-position 0

!!! Hit found (Scout): NQ at text-position 1

--> sub-scout reached node 5 (will be put in scout queue) ## End sub-scout creation

--> Scout died following edge Z from node 4 to node 0 (due to prefix loss of 3)

--> Scout died following edge N from node 3 to node 6 (due to prefix loss of 3)

--> Scout died following edge N from node 5 to node 6 (due to prefix loss of 3)

\# Created scout following edge E from node 0 to node 8

\# No scout created following edge Q from node 0 (ended in root) Primary following edge Z from node 0 to node 0

--> Scout successfully followed edge N from node 8 to node 9

!!! Hit found (Scout): ENQ at text-position 2

!!! Hit SKIPPED (Scout), since AAA is not included: NQ at

text-position 3

--> Scout successfully followed edge Q from node 9 to node 10 Primary following edge N from node 0 to node 6

Primary following edge Q from node 6 to node 7

!!! Hit found (primary): NQ at text-position 3 Found 5 peptides in protein ABZNQ.
